# Supplementary material for: HMOs Induce Butyrate Production of Faecalibacterium prausnitzii via Cross-Feeding by Bifidobacterium bifidum with Different Mechanisms for HMO Types
Source: Microorganisms. 2025 Jul 21;13(7):1705. doi: 10.3390/microorganisms13071705 (PMC12298403; doi:10.3390/microorganisms13071705)
Supplement: Supplementary file 1 [file microorganisms-13-01705-s001.zip › microorganisms-3700008-supplementary.pdf]

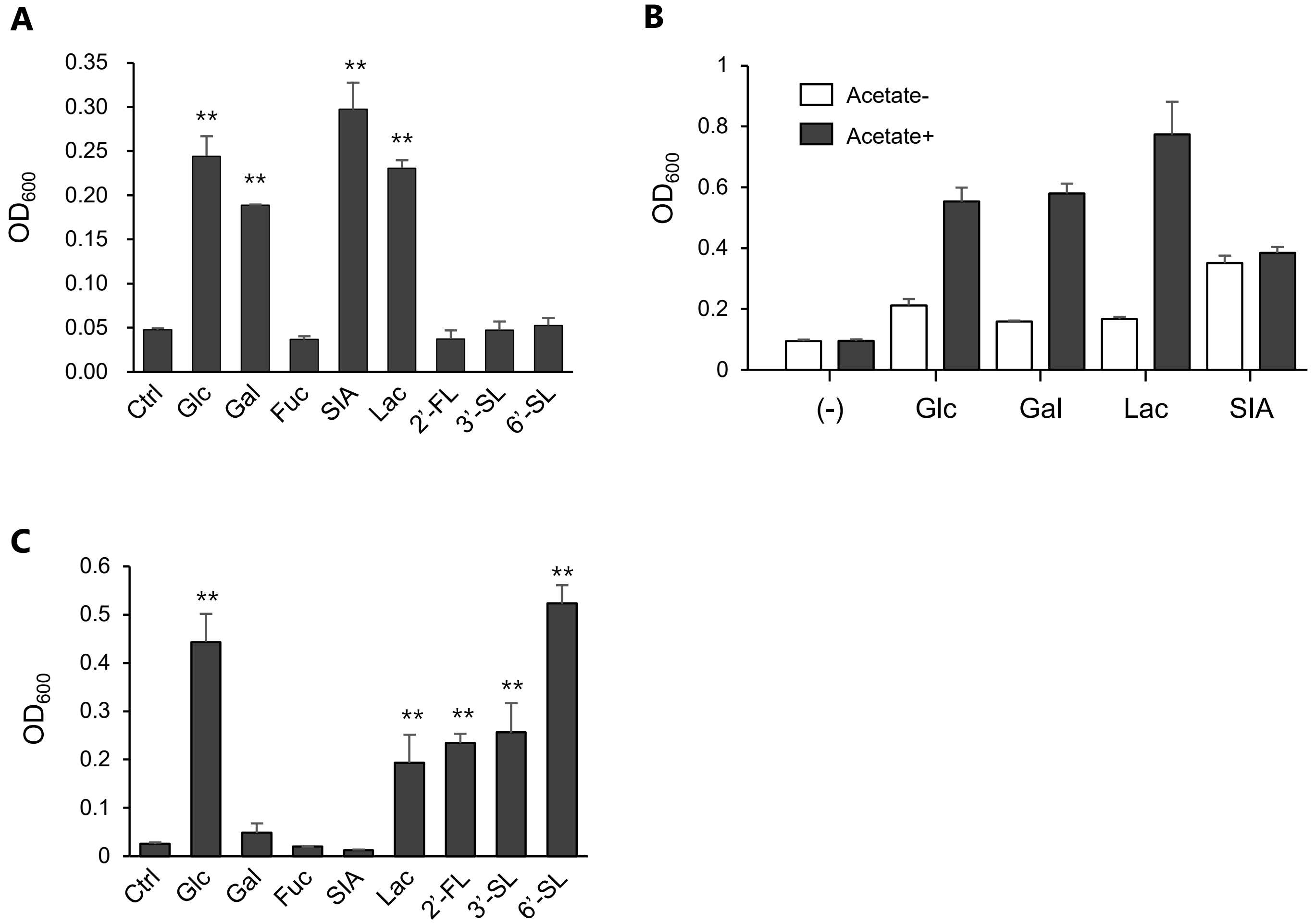

Supplementary Figure S1. Growth of *F. prausnitzii and B. bifidum* in mono-culture with individual sugars. (A) Growth of *F. prausnitzii* after 24 h of mono-culture under each sugar. (B) Growth of *F. prausnitzii* after 24 h of mono-culture under acetate and each sugar. (C) Growth of *B. bifidum* after 24 h of mono-culture under each sugar. Data are shown as the means ± standard deviation ($n = 3$). Differences were analyzed using Dunnett's test vs. Ctrl. Ctrl, control; Glc, glucose; 2′-FL, 2′-fucosyllactose; 3′-SL, 3′-sialyllactose; 6′-SL, 6′-sialyllactose. **: $p < 0.01$.
